# A Case of Odontogenic Infection by *Streptococcus constellatus* Leading to Systemic Infection in a Cogan's Syndrome Patient

**DOI:** 10.1155/2014/793174

**Published:** 2014-11-20

**Authors:** Masanobu Abe, Yoshiyuki Mori, Ryoko Inaki, Yae Ohata, Takahiro Abe, Hideto Saijo, Kazumi Ohkubo, Kazuto Hoshi, Tsuyoshi Takato

**Affiliations:** ^1^Department of Oral & Maxillofacial Surgery, University of Tokyo Hospital, 7-3-1 Hongo, Bunkyo-ku, Tokyo 113-8655, Japan; ^2^Division for Health Service Promotion, University of Tokyo, 7-3-1 Hongo, Bunkyo-ku, Tokyo 113-003, Japan

## Abstract

Odontogenic infection in immunocompromised patients tends to extend systemically beyond the oral cavity. Our case report presents a patient with sepsis due to a *Streptococcus constellatus* (*S. constellatus*) odontogenic infection in a 64-year-old-immunocompromised woman with Cogan's syndrome. She had been suffering from chronic mandibular osteomyelitis which was thought to have been caused by dental caries and/or chronic periodontitis with furcation involvement of the left mandibular first molar. We suspect that the acute symptoms of the chronic osteomyelitis due to *S. constellatus* led to the systemic infection. This infection could be accelerated by the use of a corticosteroid and an alendronate. This is the first report which represents the potential association between odontogenic infection and Cogan's syndrome.

## 1. Introduction

Odontogenic infections consist primarily of dental caries or periodontal disease. They are usually localized, but they sometimes spread systemically [1]. Many types of severe complications accompanying odontogenic infections have been reported, including cellulitis, osteomyelitis, sinusitis, thoracic empyema, mediastinitis, necrotizing fasciitis, cerebral abscess, meningitis, and septicemia [2]. The group of streptococci species known as* Streptococcus milleri* have strong virulence as pathogens, even though they reside as a part of the normal flora in the oral cavity [3].

Propagation of the infection depends on the local and systemic factors of the patient and on the virulence of the pathogen. For example, there is significant evidence that several systemic conditions, such as cardiovascular disease, diabetes mellitus, osteoporosis, and adverse pregnancy outcomes, are linked to chronic periodontal disease [4].

Cogan's syndrome is considered to be autoimmune or immune mediated in origin, supported mainly by the beneficial response to corticosteroids [5]. The diagnosis of this syndrome is mainly clinical and is based on audiovestibular symptoms, ocular inflammation, and nonreactive serologic tests for syphilis in the presence of histologically proven vasculitis. No report could be found which is describing the association between Cogan's syndrome and oral infection. We experienced the odontogenic infection in a patient with Cogan's syndrome who was diagnosed as having sepsis due to* S. constellatus* which is known as one of the oral indigenous bacterium.

## 2. Case Report

In February 2014, a 64-year-old Japanese female with a history of Cogan's syndrome was brought by ambulance to a hospital due to her high fever, chills, and dizziness. She was emergently hospitalized because of hypoxemia and cardiac insufficiency. The initial examination showed hypoxemia (SpO2 81%), tachycardia (pulse rate 130/min), and high fever (39.4°C). Her level of consciousness was I-1 (JCS) and her blood pressure was 97/56 mmHg. Echocardiography showed pulmonary hypertension (RVSP 110). A CT scan showed bilateral pulmonary congestion and obvious pulmonary artery dilatation. A blood test showed a low white blood cell count (WBC 3900, Neutrophils 80.1%), anemia of chronic disease (RBC 321 × 10^4^/*μ*L, Hb 9.7 g/dL), thrombopenia (PLT 14.7 × 10^4^/*μ*L), and increased inflammatory response (CRP 10.07 mg/dL).

The patient had been diagnosed with Cogan's syndrome in 2008, which had shown (1) vestibular disorder, (2) auditory disorder, (3) ocular inflammation, (4) aortitis, and (5) hypertrophic pachymeningitis/meningitis since 2006. She also had autoimmune hemolytic anemia (AIHA). Since symptoms (1–3) had been stable since the diagnosis, she underwent a cochlear implantation and a cataract operation. She had been taking 14 mg prednisolone for the prior seven years as the primary treatment for Cogan's syndrome and was consequently in immunocompromised status. She had been taking an alendronate (bisphosphonate) for 2 years and 9 months (from July 2010 to March 2013).

At this time, sepsis was suspected and empirical antibiotic treatment was started with i.v. meropenem (1 g every 8 h). Although the infectious focus had not been identified, the physician in charge suspected odontogenic infection as one of the candidate infectious foci, because she had been complaining of a toothache in the left mandible. Cellulitis of unknown cause in the left ankle had been observed 2 days before here hospitalization and was also considered as another candidate for the infectious focus.

To investigate the possibility of odontogenic infection, the patient was referred to our department of oral and maxillofacial surgery 5 days after her hospitalization. Gram-positive cocci (GPC) had been detected in her venous blood culture at the initial hospitalization. However, the species of the bacteria had not yet been identified. Just before the hospitalization, the pain in her left lower molar had been so severe that she had sometimes been unable to sleep. However, at her first presentation in our department, the only thing she described was uncomfortableness of the left lower molar, and no acute symptoms were observed.

As clinical findings, no inflammatory reactions such as swelling of the gingiva, discharge of pus, severe dental caries, or periodontitis were apparent in the oral cavity including the region about which the patient complained. The oral hygiene condition was quite well. Only slight percussion pain was observed in the left mandibular molars. The maximum obtainable probing depth of the left mandibular first molar and second molar was 6 mm and 4 mm, respectively. The other maximum obtainable probing depths were all 2 or 3 mm. However, Touch Test Sensory Evaluators showed hypoesthesia of the left lower lip with the complaint of numbness of the region (Vincent's symptom), which suggested the possibility of the presence of mandibular osteomyelitis.

**Figure 1:**
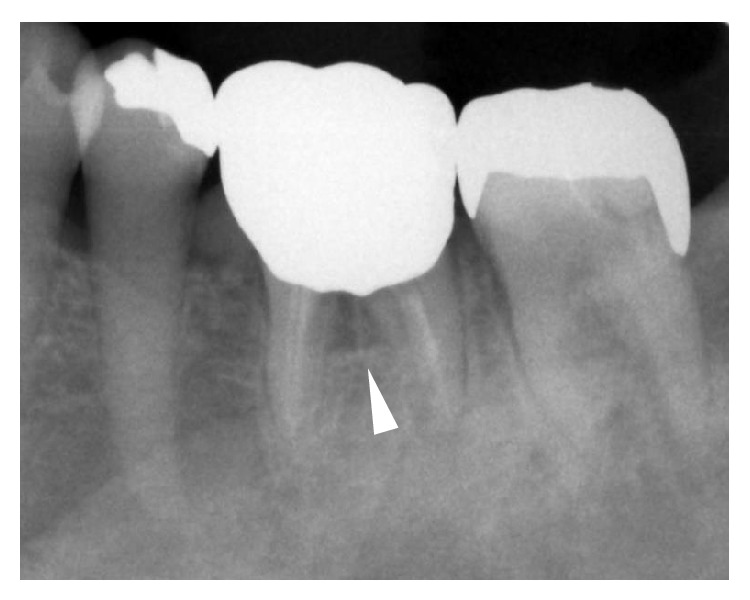
Dental X-ray indicated apparent furcation involvement of the left mandibular first molar.

**Figure 2:**
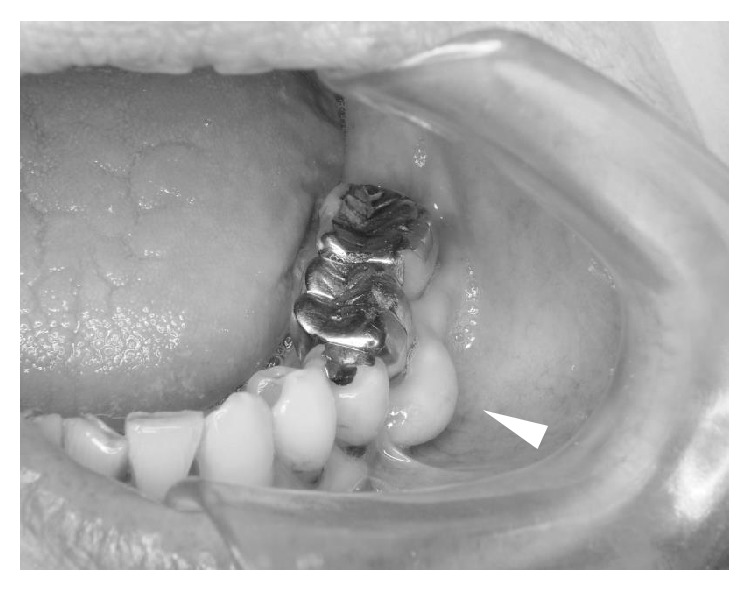
Obvious swelling of the buccal alveolar bone at the left mandibular first molar was observed. No other bone swelling was observed.

**Figure 3:**
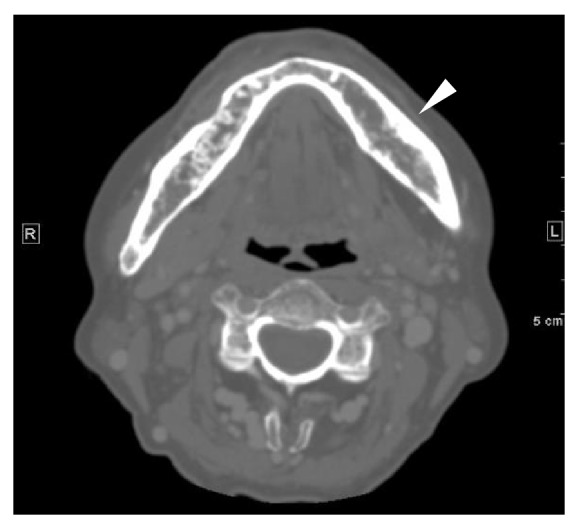
An enhanced CT scan indicated the mixture of acute and chronic osteomyelitis in the left mandible.

Pantomography and dental X-ray indicated furcation involvement of the left mandibular first molar (Figure 1). The furcation involvement in our case was classified into the Class II by the classification by Hamp and Nyman and Lindhe's classification. In addition, interestingly, marked and independent swelling of the buccal alveolar bone at the left mandibular first molar was observed (Figure 2). On the day following the patient's first visit to the oral and maxillofacial surgery department, the GPC turned out to be* S. constellatus*, which is known to be part of the normal flora in the oral cavity. This result suggested that the infectious focus was in the patient's oral cavity and prompted us to conduct a closer examination of the oral lesion. Magnetic resonance imaging (MRI) was not an option for this patient because she had undergone a cochlear implantation, but an enhanced computerized tomography (CT) scan indicated the mixture of acute and chronic osteomyelitis in the left mandible (Figure 3).

Taking all of the above-described information into consideration, we suspected that the acute symptoms of chronic osteomyelitis in her left mandible could have led to the systemic infection. The furcation involvement and/or dental caries in the left mandibular first molar was/were considered the primary focus of her current acute symptoms. Therefore, we first removed the metal crown on the left mandibular first molar. Because of the effect of the initial empiric administration of an antibiotic, no suppuration was observed, but a clear carious lesion expanding from the branch of the roots to the distal root was observed. Specimens from the soft dentin of the carious lesion were sampled for a culture-test, which revealed* methicillin-resistant Staphylococcus aureus* (MRSA) and* Enterococcus faecalis *(Group D* streptococci*). This result implied that there had been an aggressive battle between the antibiotics administered to the patient and invasive bacteria. We removed as much of the infected soft dentin of the left mandibular first molar as possible, and the molar was eventually extracted under the administration of i.v. vancomycin (1 g every 12 h) and p.o. clyndamycin (300 mg every 8 h) after the patient's general condition had recovered.

## 3. Discussion

We encountered a case of odontogenic infection by* S. constellatus* that led to systemic infection in a Cogan's syndrome patient. The patient had been suffering from chronic mandibular osteomyelitis of the mandible for a long time, and over that period, acute symptoms of osteomyelitis caused by the oral indigenous bacterium* S. constellatus* were observed. When the patient visited the oral and maxillofacial surgery department, she was already undergoing intravenous antimicrobial therapy to combat* S. constellatus* as well as anaerobes. The active infection site was thus not clear clinically in her oral cavity. However, considering that MRSA was detected in the soft dentin around the furcation involvement of her left mandibular first molar after the first empiric antibiotic therapy, we considered that the dental caries and/or chronic periodontitis of/around the left mandibular first molar was the most likely focus of the acute osteomyelitis leading to the patient's systemic infection [1, 2].

Cogan's syndrome is a chronic inflammatory disorder that most commonly affects young adults. Its clinical hallmarks are interstitial keratitis and vestibuloauditory dysfunction. Associations between Cogan's syndrome and systemic vasculitis (as well as aortitis) also exist [5]. We were unable to find any reports describing an association between Cogan's syndrome and oral disease such as periodontitis and dental caries. However, many clinical studies indicate a potential association between chronic periodontitis and the autoimmune disease rheumatoid arthritis (RA) [6].

In both RA and periodontitis, an imbalance between proinflammatory and anti-inflammatory cytokines—which are thought to be responsible for the tissue damage—is evident. Both RA and periodontitis are associated with bone destruction, mediated by inflammatory cytokines such as interleukin 1 (IL-1), tumor necrosis factor-alpha (TNF-*α*), and prostaglandin E2 [7]. Our patient had been taking a high dose of PSL for no less than 7 years as the primary treatment for Cogan's syndrome. Considering the autoimmune- or immune-mediated character of Cogan's syndrome and the fact that the patient was in long-term immunosuppressive therapy, we supposed that she was compromised enough to suffer sepsis caused by normal flora of the oral cavity. In addition, the patient's history of bisphosphonate (BP) treatment is noteworthy. When we treated her, she had been taking alendronate sodium hydrate for the prior 2 years and 9 months. Although BP therapy can provide great benefits, various side effects may develop, the most serious of which is BP-related osteonecrosis of the jaw (BRONJ). Our patient's BP treatment history might have been associated with the initiation and progression of her osteomyelitis [8].

*S. constellatus* was detected by blood culture in the patient. This bacterium is known as one of the streptococcal species of the* Streptococcus milleri* group along with* S. intermedius* and* S. anginosus*. The* S. milleri* group is a part of the normal flora in the oral cavity and the gastrointestinal tract and has been isolated from dental caries and periodontal disease [9]. Importantly, the* S. milleri* group is characterized by a tendency for abscess formation and strong pathogenesis which leads to life-threatening systemic infections. If no abscess is clinically apparent, isolation of* S. milleri* group should prompt further radiographic evaluation for abscess [3]. The presence of this group in the oral cavity predisposes to not only endodontic infections but also maxillofacial infections, which can lead to distant metastatic infections involving the lung, brain, liver, kidney, or soft tissues [10]. We observed left ankle cellulitis in the patient, and this had been suspected as another focus of septicemia before the identification of* S. constellatus*. However, considering the properties ofthese bacteria, it could be said that the ankle cellulitis itself originated from the systemic infection of* S. constellatus* [10].

This is the first report describing the odontogenic infection which extended systemically beyond the oral cavity in a Cogan's syndrome patient. Periodontal infection including furcation involvement likely plays a facilitating role in enabling the systemic dissemination of oral bacteria.
